# Polygenic risk score: use in migraine research

**DOI:** 10.1186/s10194-018-0856-0

**Published:** 2018-04-05

**Authors:** Mona Ameri Chalmer, Ann-Louise Esserlind, Jes Olesen, Thomas Folkmann Hansen

**Affiliations:** Department of Neurology, Danish Headache Center, Copenhagen University Hospital, DK-2600 Glostrup, Denmark

## Abstract

**Background:**

The latest Genome-Wide Association Study identified 38 genetic variants associated with migraine. In this type of studies the significance level is very difficult to achieve (5 × 10^− 8^) due to multiple testing. Thus, the identified variants only explain a small fraction of the genetic risk. It is expected that hundreds of thousands of variants also confer an increased risk but do not reach significance levels. One way to capture this information is by constructing a Polygenic Risk Score. Polygenic Risk Score has been widely used with success in genetics studies within neuropsychiatric disorders. The use of polygenic scores is highly relevant as data from a large migraine Genome-Wide Association Study are now available, which will form an excellent basis for Polygenic Risk Score in migraine studies.

**Results:**

Polygenic Risk Score has been used in studies of neuropsychiatric disorders to assess prediction of disease status in case-control studies, shared genetic correlation between co-morbid diseases, and shared genetic correlation between a disease and specific endophenotypes.

**Conclusion:**

Polygenic Risk Score provides an opportunity to investigate the shared genetic risk between known and previously unestablished co-morbidities in migraine research, and may lead to better and personalized treatment of migraine if used as a clinical assistant when identifying responders to specific drugs. Polygenic Risk Score can be used to analyze the genetic relationship between different headache types and migraine endophenotypes. Finally, Polygenic Risk Score can be used to assess pharmacogenetic effects, and perhaps help to predict efficacy of the Calcitonin Gene-Related Peptide monoclonal antibodies that soon become available as migraine treatment.

**Keywords:**

Migraine genetics; Genome-Wide Association Studies; Polygenic Risk Score; pleiotropy; endophenotype.

## Review

### Introduction

Migraine is a prevalent and disabling disease [1] with an incompletely understood etiopathology. The hereditary component of migraine, i.e. the proportion of individual differences explained by genetic variation in migraine, is estimated to be between 38 and 53% and is likely to arise from the combined effect of many common risk variants each with small effect sizes, thus characterizing migraine as a common complex, polygenic disease [2–4]. There is a wide range of allelic variation in human disease genes, and one common form of variation is the Single Nucleotide Polymorphism (SNP). SNPs have been valuable as genomic “markers” in the search for causal variants that influence susceptibility to common diseases, or as causal variants with marginal effect. The most common way to discover common variants is through Genome-Wide Association Studies (GWAS). A GWAS is based on the common-disease common-variant (CDCV) hypothesis and seeks to explore many SNPs randomly distributed across the human genome. A GWAS is a relatively simple way to test multiple SNPs and their contribution to disease susceptibility by comparing risk allele frequencies in cases against healthy controls [5]. To date, 38 genetic loci with common SNPs associated with migraine have been discovered [6], where the individual SNP only explains a marginal proportion of the genetic variance. Calculating the Polygenic Risk Score (PRS) is one way to assess the additive effect of several (associated) SNPs. Using a PRS calculated from sufficiently powered studies is a better way to estimate the genetic variance of the disease assessed than the individual genome-wide significant SNPs [7]. Further, some PRS methods allow researchers to assess genetic overlaps between comorbid diseases, i.e. genetic correlations, which have previously only been identified by epidemiological or clinical studies.

Our aim is here to describe the concept of the PRS approach to facilitate understanding of PRS analysis among migraine researchers with a limited expertise in molecular genetics. PRS has been studied sparsely in migraine. Thus, we use examples from neuropsychiatric disorders as they are also common brain disorders, and PRS has been widely used with great success within this field. Finally, we discuss the opportunities offered by key PRS approaches in future migraine research.

## Methods

We identified peer-reviewed studies applying polygenic methods in schizophrenia, bipolar disorder, major depressive disorder, and attention-deficit/hyperactivity disorder (ADHD) as classified by the DSM-IV using the following search terms for each of the three conditions: “Polygenic” and “risk”; “polygenic” and “analysis”; “polygenic” and “variation”; “polygenic” and “methods” in abstracts, or MESH, or text terms in Medline. We then scanned the reference lists from the selected articles for key references to find additional studies. The search was limited to English language publications no older than nine years (published from January 2009 to January 2018). Exclusion criteria were animal studies and reviews. Abstracts and titles were rated independently (two researchers: MAC and ALE). The articles were categorized into two groups: Group one included articles where the main focus in the papers was PRS, and/or papers where the PRS methods were used, and thus relevant for the review; group two included articles that did not describe or use a polygenic risk scoring method, and thus not relevant for the review. Abstracts that were relevant to migraine research included genetic risk scoring of complexly inherited neuropsychiatric traits. Abstracts on genetic scoring in all types of cancers were not included, because cancer differs markedly from that of brain disorders, such as migraine. The search yielded 146 articles; out of which 38 fulfilled the inclusion criteria and were included in the review.

## Understanding the polygenic risk score

A detailed review of the methodology of polygenic score methods is beyond the scope of this article and has been described elsewhere [7, 8]. PRS analysis allows for more genetic information to be assessed from genomic data than a simple threshold approach, such as the GWAS threshold, which conventionally uses a *p*-value threshold of 5 × 10^− 8^ to avoid issues of false positive findings due to multiple testing. The PRS approach relies on the theory that heritability, i.e. the amount of phenotypic variation explained by genetic components, of complex traits, is caused by an additive effect of multiple common gene variants with small effect sizes, a so-called polygenetic effect that is traditionally identified by GWAS. It was initially introduced as a summary score of the gene variants that are below the GWAS-significant threshold value, but the score has also been shown to be valuable when including variants that are above the GWAS threshold.

**Fig. 1:**
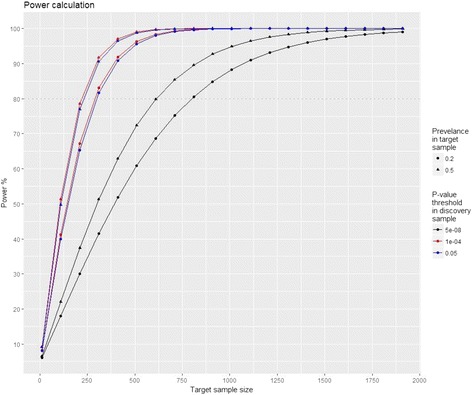
AVENGEME [7] was used to calculate the study power (y-axis) given different target sample sizes (x-axis) for migraine. For the calculation we used assumptions derived from Gormley et al.: Discovery cohort = 375.000; variance explained in the discovery sample = 0.1463; and a prevalence of 0.158. Further, we assumed that the fraction of NULL SNPs is 0.95 and that the outcome is binary. The effects from Gormley et al. are used to weigh the SNPs for calculating the PRS. We present six curves representing two different prevalences of migraine in the target sample (circle = 0.2 and triangle = 0.5) given three different P_T_ in the discovery sample (black = 5 × 10^− 8^, red = 1 × 10^− 4^, and blue = 0.05

To construct a PRS, an initial GWAS is done which is considered the discovery sample. In an independent sample with GWAS data, denoted the target sample, the PRS is calculated for each individual by adding up the risk alleles weighted by their odds ratios from the discovery sample. It is then possible to evaluate the prediction value of PRS using e.g. the coefficient of determination from the regression analysis, also expressed as R^2^ [9]. This can be done using different significance thresholds (P_T_) of the data from the discovery sample, thereby testing whether including more information, i.e. SNPs, increases the power of prediction. For successful construction of the PRS four prerequisites has been suggested: The target and discovery samples must be large (*n* > 2000 [7]); the discovery sample must be at least as large as the target sample; the phenotype investigated must be relatively homogeneous; and the level of genetic variation explained by common variants must be high [7]. We used data from the latest migraine meta-analysis as discovery sample [6] to conduct power calculations to estimate the number of samples required to derive a clinically useful predictor for migraine risk. We used the statistical R package AVENGEME [7, 10, 11] and provided a power calculation based on two different migraine prevalences in the target sample and for three different P_T_, see fig. 1. A sample size of 300, given P_T_ of 1 × 10^− 4^ and 0.05, provides a study power of 80%, however, by using the genome-wide P_T_ (5 × 10^− 8^) a sample size of more than 800 samples is needed, assuming a migraine prevalence of 0.2. Large discovery cohorts are needed to obtain decent power in smaller target samples. This calls for large collaborations, e.g. UK Biobank and International Headache Genetics Consortium.

## Lessons from genetic studies of neuropsychiatric disorders

Since most neuropsychiatric disorders are common brain disorders, we sought it relevant to retrieve inspiration to migraine PRS studies from here. There have been several studies on neuropsychiatric disorders using the PRS to assess: Prediction of disease status in case-control studies, shared genetic correlation between co-morbid diseases, and shared genetic correlation between a disease and specific endophenotypes. We shall highlight key concepts of PRS analysis from neuropsychiatric genetics, focusing on these polygenic approaches.

### Prediction of case-control status

Purcell et al. prompted the use of nominal associated SNPs in neuropsychiatric research in 2009 [9]. By creating a genetic risk score, the authors showed that the power of a large GWAS could be applied to smaller sample sizes and could predict case-control status. Primarily, this was an investigation of a single disease (schizophrenia) where the SNPs derived from the discovery sample were tested in an independent schizophrenia replication sample. The authors found that SNPs with significance level P_T_ < 0.05 were correlated with schizophrenia in the target sample (*P* = 9 × 10^− 19^) and explained approximately 3% of the genetic variance.

Table 1 gives an overview of the studies that have since investigated the prediction of case-control status (risk prediction) using PRS analysis. The studies show consistent patterns across different phenotypes with significant disease prediction capacity but low ability to explain variance in genetic liability (between 0.2 and 5%).

**Table 1:** Prediction of case control status using Polygenic Risk Score

Reference	Discovery sample	Target sample	Outcome
Ruderfer et al. [51]	2794 cases (SCZ) and 2976 controls	334 cases (SCZ) and 360 controls	Variance explained by SCZ PRS was 5%. The PRS was higher in cases than controls. Population stratification did not influence the outcome.
Chang et al. [52]	6989 cases (NHS)	3 of the 4 NHS-GWAS [53] sub-studies were used as training sets	PRS was estimated by 3 different approaches: internal whole-genome scoring and two external PRS weighting algorithms from independent samples. The 3 PRS approaches explained 0.2% of the variance in depressive symptoms.
Kauppi et al. [20]	9146 cases (SCZ) and 12,111 controls	63 cases (SCZ) and 118 controls	PRS was significantly higher in patients than controls, and a higher PRS was associated with dysfunction of frontal lobe activation during work-memory related tasks.

*SCZ* Schizophrenia, *NHS* Nurses’ Health Study, *GWAS* Genome Wide Association Study

### Investigation of pleiotropy: The shared genetic risk between distinct phenotypes

The ability of a genetic variant to associate with more than one phenotype is referred to as pleiotropy. The pleiotropic effect of the SNPs may indicate that different diseases are genetically correlated [12, 13]. Polygenic methods can be used to test the genetic correlation between two distinct diseases. As an example, a polygenic score derived from a discovery sample from consortium data in schizophrenia was applied to seven different target samples: One bipolar cohort and six other non-neuropsychiatric cohorts [9]. The schizophrenia PRS predicted bipolar disorder status but had no correlation with non-neuropsychiatric traits. Thus, the study supported epidemiological results correlating the two diseases [14]. The variance of genetic liability to bipolar disorder explained by the polygenic score was small (R^2^ = 0.019), but still a significant portion of the total SNP heritability could be explained by the schizophrenia PRS [9]. This approach gained further support from studies conducted by the cross disorder group of the Neuropsychiatric Genomics Consortium and it found overlapping genetic loci, i.e. pleiotropy, for childhood-onset diseases (ADHD and autism) and adolescent/adult on-set diseases (bipolar disorder, major depressive disorder, and schizophrenia) [15]. Again, bipolar disorder and schizophrenia were found to correlate, but also the status of autism spectrum disorder could be predicted by polygenic scores from both schizophrenia and bipolar disorder. These studies have successfully shown that PRS may identify pleiotropy.

PRS may also be used to identify a shared genetic background of unknown co-morbid traits. Powell et al. recently showed that an aggregate of common variants conferring risk of schizophrenia and bipolar disorder may underlie creativity in artists [16].

Studies investigating pleiotropy, using PRS are indexed in Table 2. PRS is a significant predictor of pleiotropy, but the variance of genetic liability is still low, ranging from 0.1% to 2.1%.

**Table 2:** Prediction of pleiotropy using Polygenic Risk Score

Reference	Discovery sample	Target sample	Outcome
Goes et al. [54]	2196 cases (BP) and 8148 controls	The PGC SCZ results	The PRS analysis showed a genetic overlap between BP with mood-incongruent psychotic features and SCZ.
Demirkan et al. [55]	1738 cases (MDD) and 1802 controls	2286 cases (MDD and anxiety) and 1205 controls	MDD-PRS explained up to 0.7% of the variance in depression in the study sample. The MDD-PRS was associated with anxiety and explained up 2.1% of the anxiety variance in the study population.
Peyrot et al. [56]	7544 cases (MDD) and 7754 controls	1645 cases (MDD) and 340 controls	Persons with both high MDD-PRS and history of childhood trauma are at risk for developing MDD in adolescence.
Neuropsychiatric GWAS Consortium Bipolar Disorder Working Group [57]	7481 cases (BP) and 9250 controls Replication study: 4493 cases (BP) and 42,542 controls	675 cases (BP) and 1297 controls	SCZ-PRS contributes to the risk of bipolar disorder.
Ruderfer et al. [58]	9369 cases (SCZ) and 8723 controls	10,410 cases (BP) and 10,700 controls	There is a significant correlation between a BP-PRS and the clinical dimension of mania in SCZ patients. BP-PRS was associated with only the manic factors in SCZ patients, the association between BP-PRS and mania was largest at the high end of the mania distribution. BP-PRS explained 2% of the variance.
Ikeda et al. [59]	236 cases (METH-dependent), 864 controls	560 cases (SCZ), 548 controls	There was a shared genetic risk between METH-induced psychosis and SCZ.
Solovieff et al. [60]	3322 cases (SCZ), 3587 controls [9]	845 cases (PTSD), 1693 controls	There was an association between BP-PRS and PTSD severity.
Byrne et al. [61]	6324 cases (MDD), 6678 controls	4 different MDD and PDD target samples were used: 2104 PPD cases, 3149 MDD cases, 9447 PPD screened controls, 3468 MDD screened controls	BP-PRS explained 0.1% of the post-partum-depression variance.
Ferentinos et al. [62]	Cohort from the PGC MDD and BP mega-analysis [57, 63]	1966 cases from the RADIANT studies (MDD)	MDD-PRS predicted depression episodicity, and episodicity was better predicted with MDD-PRS than with BP-PRS.
Wiste et al. [64]	Meta-analysis from PGC 2011 (BP) [57]	1274 cases (MDD) Further replication: 992 cases (MDD), 585 cases (MDD)	BP polygenic genetic load was associated with bipolar-like presentation in MDD. The results were, however, inconclusive since they were not replicatable. BP-PRS explained 0.8%–1.1% of the variance in depression traits.
Musliner et al. [65]	Results of the combined GWAS of MDD by the PGC [63]	HRS target dataset, 8761 participants.	Stressful life events did not mediate or confound the association between MDD-PRS and depressive symptoms, however; MDD-PRS and stressful life events were independent, significant predictors of depressive symptoms and MDD-PRS explained less than 1% of the variance in depressive symptoms.
Derks et al. [66]	8690 cases (SCZ), 11,831 controls	314 cases (SCZ), 148 controls	No significant correlation between SCZ-PRS and quantitative domains of SCZ symptoms in SCZ cases and controls.
Mullins et al. [67]	7 discovery datasets (MDD, BP)	4 validation/target sets (3 sets for suicide attempt, 1 from suicide ideation).	MDD-PRS predicted suicidal ideation. There was no polygenic association between suicide attempt and suicidal ideation, suggesting that suicide attempts and suicidal ideation are not part of the same spectrum, thus the tendency to act on suicidal thoughts may have another proponent than suicidal ideation.

*BP* Bipolar Disorder, *SCZ* Schizophrenia, *MDD* Major Depressive Disorder, *METH* Methamphetamine, *PTSD* Post Traumatic Stress Disorder, *PGC* Neuropsychiatric Genomics Consortium, *GWAS* Genome Wide Association Study, *HRS* Health and Retirement Study

### Investigation of polygenic risk score and endophenotypes

Many complex genetic diseases are heterogeneous with regards to e.g. symptomatology and age of onset. The heterogeneity of these diseases may reflect the multifactorial and polygenic origin of the disease. Neuropsychiatric disorders and other neurological diseases like migraine and other headache diseases are clinically defined phenotypes that are not supported by measurable biomarkers. An endophenotype is a genetic-epidemiological concept which aims to group behavioral symptoms into more clear-cut phenotypes of a heritable disease. Endophenotypes are assumed to detect the genetic risk of disease regardless of the manifestation of the disease phenotype, and may, therefore, define the biological etiology better than the clinical diagnosis [17].

Endophenotypes have been widely studied in neuropsychiatric research [17], in other brain diseases, e.g. Alzheimer’s disease [18] as well as in other hereditary diseases e.g. cystic fibrosis [19]. The definition and criteria for endophenotypes used in neuropsychiatric research are listed in Table 3.

**Table 3:** The criteria for endophenotypes (adapted from Gottesman et al. [17])

1	The endophenotype is associated with illness in the population
2	The endophenotype is heritable
3	The endophenotype is primarily state independent (manifests in an individual whether or not illness is active)
4	Within families, the endophenotype and illness co-segregate
5	The endophenotype found in affected family members are found in non-affected family members at a higher rate than in the general population

An endophenotype of schizophrenia, dysfunction of frontal lobe activation during work-memory related tasks, has been associated with higher PRS for schizophrenia [20]. These findings are further supported by studies on healthy patients using both structural and functional brain imaging in which the schizophrenia-derived PRS showed to associate with the differences in activity during verbal testing [21, 22]. Likewise, another study found a non-significant trend in the differentiation of bipolar disorder with and without psychosis using the schizophrenia-derived PRS [23]. Further examples of studies using PRS to dissect endophenotypes are listed in Table 4.

**Table 4:** Studies using Polygenic Risk Score to dissect endophenotypes

Reference	Discovery sample	Target sample	Outcome
Nievergelt et al. [68]	Based on data downloaded from the PGC website for BP, MDD and SCZ	940 cases (PTSD), 2554 controls	PRS calculated from GWAS of BD could significantly predict PTSD in U.S. marine soldiers, while PRS from a SCZ and MDD GWAS could not predict PTSD in the U.S. marines.
Middeldorp et al. [69]	13,835 (personality traits)	1) 1738 cases (MDD), 1802 controls. 2) 2101 cases (BP), 3280 controls	Shared polygenic risk factors between neuroticism and MDD and between BP and extraversion. The explained variance of MDD and BP was 0.1%.
Terwisscha van Scheltinga et al. [70]	8690 cases (SCZ), 11,831 controls	152 cases (SCZ), 142 controls	SCZ-PRS was associated with total brain volume (measured by fMRI). PRS was specifically associated with reduced white matter volume, and did not explain variance in grey matter volume. The higher SCZ-PRS the smaller total brain volumes. Disease status was predicted by PRS.
Whalley et al. [71]	Based on data downloaded from the PGC website for BP and MDD	70 cases (unaffected, but at familial risk of mood disorder), 62 controls	Correlation between high polygenic risk for MDD and reduced white matter integrity. No association with BP-PRS and white matter volume.
Walton et al. [72]	3322 cases (SCZ), 3587 controls	255 cases (SCZ)	Increased polygenic risk for SCZ is associated with neural inefficiency in the dorsolateral prefrontal cortex.
Hall et al. [73]	Based on data downloaded from the PGC website for SCZ, BP and MDD	271 cases (SCZ or psychotic BP), 128 controls	There is a genetic overlap between SCZ loci and gamma oscillation and between BP loci and P3 amplitude. Patients with a high SCZ-PRS had reduced gamma response, and patients with a high BP-PRS had smaller P3 amplitude. SCZ-PRS explained 4% of the variance in gamma oscillation phenotype and BP-PRS explained 3% of the variance in P3 amplitude phenotype.
Holmes et al. [74]	9240 cases (MDD), 9519 controls	438 healthy cases	There is a significant association between increasing polygenic burden for MDD and reduced cortical thickness in the left mPFC. MDD-PRS accounts for 4–9% of the phenotypic variance of the amygdala prefrontal cortex thickness.
Whalley et al. [22]	7481 cases (BD), 9250 controls	For genetic information: 87 cases (BD), 71 controls. For fMRI data: 73 cases (BD), 52 controls	High BD-PRS was associated with high activity in limbic regions.

*PGC* Neuropsychiatric Genomics Consortium, *PTSD* Post Traumatic Stress Disorder, *BP* Bipolar Disorder, *SCZ* Schizophrenia, *MDD* Major Depressive Disorder, *GWAS* Genome Wide Association Study, *fMRI* Functional Magnetic Resonance Imaging, *P3* Event related potential 3, *mPFC* Medial Prefrontal Cortex

Furthermore, PRS analysis has been used to probe different responses to pharmacological treatment as aberrant drug responses may be proxies or even regarded as endophenotypes. A recent study found that PRS derived from a meta-analysis of three genome-wide pharmacogenetic studies explained 0.5%–1% of the variance in antidepressants-response in patients with major depressive disorder [24]. Another study used PRS derived from bipolar disorder to investigate whether a lack of response to antidepressants could be explained by a high PRS for bipolar disorder. The study did not show an association between increasing PRS and lack of response to antidepressants [25]. This shows that by investigating treatment response using PRS, important and interesting research questions may be answered.

## Factors influencing polygenic risk score performance

It should be noted that the performance of the PRS is influenced by several parameters such as the underlying genetic architecture of the disease in terms of the number of causal variants and whether these have an additive effect, the effect sizes of individual causal variants, and allele frequency at the causal variants. As an example, a larger discovery sample is necessary if the genetic architecture consists of many low frequent variants with small effect sizes, as opposed to a genetic architecture that has fewer frequent variants with relatively high effect sizes. Thus, the PRS performance relies on the sample size; by increasing the discovery sample, the variance explained increases, which further increases the accuracy of the PRS for each individual. Furthermore, it has been estimated that when a target sample reaches ~ 2000 cases there should be sufficient power to detect a variance that is different from zero [7, 8]. Other factors that may influence PRS performance may be the heterogeneity of the phenotype, which paradoxically is often compromised in GWAS studies in the need for large sample sizes and better prediction power.

It is important that the discovery and target sample are independent. Thus, patients of the same ethnicity as the target sample are often excluded from the discovery sample to avoid an overestimation of the effects of the PRS.

## Suggested application of polygenic risk score analysis to migraine research

In this review we have described a method to explore the genetic architecture of common complex brain disorders. As migraine and other headache disorders resemble neuropsychiatric disorders on the complexity, the polygenetic nature, and both being common brain disorders, we have introduced PRS analysis by summarizing experiences from studies of neuropsychiatric disorders. As large migraine GWAS datasets are now available [6] it is now possible to apply polygenic methods in migraine research.

### Investigation of pleiotropy in migraine

Most studies of shared genetics have been investigated in bi- and multivariate twin model studies. These studies were hampered by the need for large twin cohorts with two or more traits of interest. A great opportunity is therefore offered by PRS analysis which may confirm these findings, and enable further investigation at a genotype level. Two migraine studies using a PRS based on small migraine GWAS datasets have already been performed. These studies compared the PRS in migraine and two important migraine co-morbidities; depression and stroke. Ligthart et al. [26] found genetic components shared between migraine and major depressive disorder (MDD). The PRS derived from GWAS on MDD could significantly predict the comorbid MDD and migraine phenotype (*P* = 0.0015), but the MDD PRS could not predict migraine without comorbid MDD (*P* = 0.058). The correlation between migraine and ischemic stroke has been investigated using data from the International Headache Genetics Consortium’s migraine meta-analysis from 2013 (discovery sample), which was applied to a sample consisting of patients with stroke (target sample) [27]. The study found genetic risk factors shared between migraine without aura (MO) and large arterial stroke (*p* = 6.4 × 10^− 28^) as well as between MO and cardio-embolic stroke (*p* = 2.7 × 10^− 20^). Recently, a study in BioRxiv by Antilla et al. [28] suggested a limited sharing of genetic risk between neurological and psychiatric disorders in general. Although, migraine was significantly correlated with ADHD (r_*g*_ = 0.26, *p* = 8.81 × 10^− 8^), Tourette Syndrome (r_*g*_ = 0.19, *p* = 1.80 × 10^− 5^), and MDD (r_*g*_ = 0.32, *p* = 1.42 × 10^− 22^), suggesting that migraine may share genetic risk with these disorders.

These are indeed interesting findings, and the latest and largest migraine GWAS presents an opportunity to confirm these findings as well as to test pleiotropy for other known migraine co-morbidities, e.g. autoimmune diseases [29], thyroid diseases [30], pain disorders [31], fibromyalgia [32], and sleep [33]. Another known migraine co-morbidity is endometriosis [34–36] and, very interestingly, a study suggested that migraine and endometriosis are genetically correlated (r_*g*_ = 0.27, 95% CI: 0.06–0.47). Furthermore, the relation between migraine and personality traits may also be tested [37, 38]. PRS may lead to better understanding of the disease and thus enable better choice of treatment for migraine patients. One can even imagine using PRS as a clinical assistant when choosing prophylactic drugs, e.g. patients who have a shared genetic component between migraine and depression may profit better from antidepressants than others. A shared genetic component may also prompt screening for depression in migraine patients with unrecognized depression.

### Investigation of migraine and endophenotypes in migraine

Migraine is a highly heterogeneous disorder. The frequency, severity, and triggering of attacks as well as the pharmacological effect of migraine drugs vary between patients. Defining migraine endophenotypes may narrow down the broad clinical phenotype into more homogeneous and pathophysiological relevant phenotypes; facilitate clinical trials; and possibly increase power to detect the putative genetic correlations. Menstrual relation [39] and premonitory symptoms [40] are previously described migraine endophenotypes. PRS analysis may identify more endophenotypes in migraine. Perhaps menstrual migraine can be characterized by applying the PRS from other menstrual cycle associated conditions such as endometriosis [36]. Also, it would be interesting to investigate whether migraine without aura and migraine with aura are endophenotypes or genetically distinct disorders. Keeping in mind, that an important property of endophenotypes is their heritable nature (Table 3), it may interesting to use PRS to analyze the relation between migraine and tension-type headache [41]; migraine and secondary headaches (particularly chronic post-traumatic headache); chronic and episodic migraine, and to investigate whether proneness to medication overuse is an endophenotype. A recent study investigating 12 migraine-associated SNPs and drug response found an association between increasing genetic load of migraine and the effect of triptans with odds ratios of treatment success from 1.3–2.6 (*P* < 0.05) depending on single SNPs or a genetic load of the 12 migraine-associated SNPs [42]. This encouraging finding merits further investigation using PRS analysis. It is relevant to investigate pharmacogenetic effects because a third of patients with migraine do not respond well to triptans and many more have no or poor response to prophylactic medication [43]. Perhaps PRS analysis can also be used to predict efficacy of the Calcitonin Gene-Related Peptide (CGRP) monoclonal antibodies that soon become available, presumably at a high cost.

### Prediction of case-control status in migraine

Clinical disease prediction of migraine relies exclusively upon existing classification criteria (the International Classification of Headache, third edition [44]) as there are no available objective diagnostic methods to assess migraine status in patients. Frequent migraine attacks may be associated with several cerebral disorders such as arteriovenous malformations [45], mitochondrial encephalopathies [46], and cerebral arteriopathy with subcortical infarcts and leukoencephalopathy (CADASIL) [47, 48]. Further, migraine-like attacks may be triggered by different vascular events such as cerebral infarction, cervical-artery dissection, or cerebral venous thrombosis. In the latter cases, migraine attacks are symptomatic of the underlying pathology, and symptomatic migraine attacks are often difficult to distinguish from a primary migraine disorder. PRS may assist in predicting migraine disease status in these complex cases and assist in understanding whether the mechanisms of symptomatic migraine attacks are different from those of primary migraine attacks. Additionally, PRS analysis may help to assess whether patients with organic cerebral disorders have a lower threshold than others for developing migraine attacks. Given a proper prediction power, the PRS may even assist in the migraine diagnosis [49]. A study from the Norfolk Island [50] showed a higher PRS score for patients with migraine than controls (*P* = 0.0016); and that a high PRS score resulted in a 3.1-fold increased risk of migraine. This is a very interesting finding, and the latest migraine GWAS presents an opportunity to confirm these findings.

## Conclusion

PRS analyses have shown successful progress in the research of neuropsychiatric disorders and may inspire migraine research to understand more about the genetic underpinnings of migraine. PRS may be useful in the investigation of shared genetic risk with comorbidities, in studying the relation between primary headache disorders and their sub-forms, and to personalize migraine treatment.

## Abbreviations

ADHD: Attention deficit/hyperactivity disorder
CDCV: Common disease common variant
GWAS: Genome-Wide Association Study
MO: Migraine without aura
PRS: Polygenic Risk Score
SNP: Single Nucleotide Polymorphism

## References

[1] Stewart WF, Shechter A, Lipton RB (1994). Migraine heterogeneity. Disability, pain intensity, and attack frequency and duration. Neurology.

[2] Russell MB, Olesen J (1995). Increased familial risk and evidence of genetic factor in migraine. BMJ.

[3] Mulder EJ, Van Baal C, Gaist D (2003). Genetic and environmental influences on migraine: a twin study across six countries. Twin Res.

[4] Russell MB, Iselius L, Olesen J (1995). Inheritance of migraine investigated by complex segregation analysis. Hum Genet.

[5] Bush WS, Moore JH (2012) Chapter 11: genome-wide association studies. PLoS Comput Biol 8. 10.1371/journal.pcbi.1002822. 10.1371/journal.pcbi.1002822 PMC3531285 23300413

[6] Gormley P, Anttila V, Winsvold BS (2016). Corrigendum: meta-analysis of 375,000 individuals identifies 38 susceptibility loci for migraine. Nat Genet.

[7] Dudbridge F (2013). Power and predictive accuracy of polygenic risk scores. PLoS Genet.

[8] Wray NR, Lee SH, Mehta D (2014). Research review: polygenic methods and their application to psychiatric traits. J Child Psychol Psychiatry.

[9] Purcell SM, Wray NR, Stone JL (2009). Common polygenic variation contributes to risk of schizophrenia and bipolar disorder. Nature.

[10] Palla L, Dudbridge F (2015). A fast method that uses polygenic scores to estimate the variance explained by genome-wide marker panels and the proportion of variants affecting a trait. Am J Hum Genet.

[11] Dudbridge F, Pashayan N, Yang J (2018). Predictive accuracy of combined genetic and environmental risk scores. Genet Epidemiol.

[12] Sivakumaran S, Agakov F, Theodoratou E (2011). Abundant pleiotropy in human complex diseases and traits. Am J Hum Genet.

[13] Stower H (2012). Human genetics: pleiotropic mutations. Nat Rev Genet.

[14] Fuller Torrey E (1999). Epidemiological comparison of schizophrenia and bipolar disorder. Schizophr Res.

[15] (2013) Identification of risk loci with shared effects on five major psychiatric disorders: a genome-wide analysis. Lancet 381:1371–9. doi: 10.1016/S0140-6736(12)62129-1. 10.1016/S0140-6736(12)62129-1 PMC3714010 23453885

[16] Power RA, Steinberg S, Bjornsdottir G (2015). Polygenic risk scores for schizophrenia and bipolar disorder predict creativity. Nat Neurosci.

[17] Gottesman II, Gould TD (2003). The endophenotype concept in psychiatry: etymology and strategic intentions. Am J Psychiatry.

[18] Reitz C, Mayeux R (2009). Endophenotypes in normal brain morphology and Alzheimer’s disease: a review. Neuroscience.

[19] Stanke F, Hedtfeld S, Becker T, Tümmler B (2011). An association study on contrasting cystic fibrosis endophenotypes recognizes KRT8 but not KRT18 as a modifier of cystic fibrosis disease severity and CFTR mediated residual chloride secretion. BMC Med Genet.

[20] Kauppi K, Westlye LT, Tesli M et al (2014) Polygenic risk for schizophrenia associated with working memory-related prefrontal brain activation in patients with schizophrenia and healthy controls. Schizophr Bull. 10.1093/schbul/sbu152. 10.1093/schbul/sbu152 PMC4393689 25392519

[21] Whalley HC, Hall L, Romaniuk L (2015). Impact of cross-disorder polygenic risk on frontal brain activation with specific effect of schizophrenia risk. Schizophr Res.

[22] Whalley HC, Papmeyer M, Sprooten E (2012). The influence of polygenic risk for bipolar disorder on neural activation assessed using fMRI. Transl Psychiatry.

[23] Hamshere ML, O’Donovan MC, Jones IR (2011). Polygenic dissection of the bipolar phenotype. Br J Psychiatry.

[24] (2013) Common genetic variation and antidepressant efficacy in major depressive disorder: a meta-analysis of three genome-wide pharmacogenetic studies. Am J Psychiatry 170:207–17. doi: 10.1176/appi.ajp.2012.12020237. 10.1176/appi.ajp.2012.12020237 PMC10416089 23377640

[25] Tansey KE, Guipponi M, Domenici E (2014). Genetic susceptibility for bipolar disorder and response to antidepressants in major depressive disorder. Am J Med Genet B Neuropsychiatr Genet.

[26] Ligthart L, Hottenga J-J, Lewis CM (2013). Genetic risk score analysis indicates migraine with and without comorbid depression are genetically different disorders. Hum Genet.

[27] Malik R, Freilinger T, Winsvold BS et al (2015) Shared genetic basis for migraine and ischemic stroke: a genome-wide analysis of common variants. Neurology. 10.1212/WNL.0000000000001606. 10.1212/WNL.0000000000001606 PMC4451048 25934857

[28] Anttila V, Finucane H, Walters R, et al (2016) Analysis of Shared Heritability in Common Disorders of the Brain multiple distinct brain phenotypes ( 12–14 ). Recently, genome-wide association studies ( GWAS ). doi: 10.1101/048991. doi: Anttila, V., Finucane, H., Walters, R., Bras, J., Duncan, L., Lee, P.H., Turley, P., Consortium, I., 2016. Analysis of Shared Heritability in Common Disorders of the Brain multiple distinct brain phenotypes ( 12–14 ). Recently, genome-wide association studies ( GWAS ). doi:10.1101/048991.

[29] Le H, Tfelt-Hansen P, Russell MB (2011). Co-morbidity of migraine with somatic disease in a large population-based study. Cephalalgia.

[30] Hagen K, Bjøro T, Zwart JA (2001). Low headache prevalence amongst women with high TSH values. Eur J Neurol.

[31] Hagen K, Einarsen C, Zwart J-A (2002). The co-occurrence of headache and musculoskeletal symptoms amongst 51 050 adults in Norway. Eur J Neurol.

[32] Vij B, Whipple MO, Tepper SJ et al (2015) Frequency of migraine headaches in patients with fibromyalgia. Headache. 10.1111/head.12590. 10.1111/head.12590 25994041

[33] Dosi C, Figura M, Ferri R, Bruni O (2015). Sleep and headache. Semin Pediatr Neurol.

[34] Ferrero S, Pretta S, Bertoldi S (2004). Increased frequency of migraine among women with endometriosis. Hum Reprod.

[35] Tietjen GE, Conway A, Utley C (2006). Migraine is associated with menorrhagia and endometriosis. Headache.

[36] Nyholt DR, Gillespie NG, Merikangas KR (2009). Common genetic influences underlie comorbidity of migraine and endometriosis. Genet Epidemiol.

[37] Huber D, Henrich G (2003). Personality traits and stress sensitivity in migraine patients. Behav Med.

[38] Pompili M, Di Cosimo D, Innamorati M (2009). Psychiatric comorbidity in patients with chronic daily headache and migraine: a selective overview including personality traits and suicide risk. J Headache Pain.

[39] Schurks M, Rist PM, Kurth T (2010). Sex hormone receptor gene polymorphisms and migraine: a systematic review and meta-analysis. Cephalalgia.

[40] Goadsby PJ, Charbit AR, Andreou AP (2009). Neurobiology of migraine. Neuroscience.

[41] Russell MB, Levi N, Kaprio J (2007). Genetics of tension-type headache: a population based twin study. Am J Med Genet Part B Neuropsychiatr Genet.

[42] Christensen AF, Esserlind A-L, Werge T (2016). The influence of genetic constitution on migraine drug responses. Cephalalgia.

[43] Diener H-C, Limmroth V (2001). Advances in pharmacological treatment of migraine. Expert Opin Investig Drugs.

[44] (2018) Headache Classification Committee of the International Headache Society (IHS) The International Classification of Headache Disorders, 3rd edition. Cephalalgia 38:1–211. doi: 10.1177/0333102417738202. 10.1177/0333102417738202 29368949

[45] GW B (1984) Intracranial arteriovenous malformation and migraine. Cephalalgia. 10.1046/j.1468-2982.1984.0403191.x 6498934

[46] Pavlakis SG, Phillips PC, DiMauro S (1984). Mitochondrial myopathy, encephalopathy, lactic acidosis, and strokelike episodes: a distinctive clinical syndrome. Ann Neurol.

[47] Vérin M, Rolland Y, Landgraf F (1995). New phenotype of the cerebral autosomal dominant arteriopathy mapped to chromosome 19: migraine as the prominent clinical feature. J Neurol Neurosurg Psychiatry.

[48] Chabriat H, Vahedi K, Bousser MG (1995). Clinical spectrum of CADASIL: a study of 7 families. Lancet.

[49] Foley SF, Tansey KE, Caseras X (2017). Multimodal brain imaging reveals structural differences in Alzheimer’s disease polygenic risk carriers: a study in healthy young adults. Biol Psychiatry.

[50] Rodriguez-Acevedo AJ, Ferreira MA, Benton MC (2015). Common polygenic variation contributes to risk of migraine in the Norfolk Island population. Hum Genet.

[51] Ruderfer DM, Kirov G, Chambert K, et al (2011) A family-based study of common polygenic variation and risk of schizophrenia. Mol Psychiatry 16:887–8. doi: 10.1038/mp.2011.34. 10.1038/mp.2011.34 PMC4189087 21483432

[52] Chang S-C, Glymour MM, Walter S (2014). Genome-wide polygenic scoring for a 14-year long-term average depression phenotype. Brain Behav.

[53] Cornelis MC, Monda KL, Yu K (2011). Genome-wide meta-analysis identifies regions on 7p21 (AHR) and 15q24 (CYP1A2) as determinants of habitual caffeine consumption. PLoS Genet.

[54] Goes FS, Hamshere ML, Seifuddin F (2012). Genome-wide association of mood-incongruent psychotic bipolar disorder. Transl Psychiatry.

[55] Demirkan A, Penninx BWJH, Hek K (2011). Genetic risk profiles for depression and anxiety in adult and elderly cohorts. Mol Psychiatry.

[56] Peyrot WJ, Milaneschi Y, Abdellaoui A (2014). Effect of polygenic risk scores on depression in childhood trauma. Br J Psychiatry.

[57] (2011) Large-scale genome-wide association analysis of bipolar disorder identifies a new susceptibility locus near ODZ4. Nat Genet 43:977–83. doi: 10.1038/ng.943. 10.1038/ng.943 PMC3637176 21926972

[58] Ruderfer DM, Fanous AH, Ripke S (2014). Polygenic dissection of diagnosis and clinical dimensions of bipolar disorder and schizophrenia. Mol Psychiatry.

[59] Ikeda M, Okahisa Y, Aleksic B (2013). Evidence for shared genetic risk between methamphetamine-induced psychosis and schizophrenia. Neuropsychopharmacology.

[60] Solovieff N, Roberts AL, Ratanatharathorn A (2014). Genetic association analysis of 300 genes identifies a risk haplotype in SLC18A2 for post-traumatic stress disorder in two independent samples. Neuropsychopharmacology.

[61] Byrne EM, Carrillo-Roa T, Penninx BWJH (2014). Applying polygenic risk scores to postpartum depression. Arch Womens Ment Health.

[62] Ferentinos P, Rivera M, Ising M (2014). Investigating the genetic variation underlying episodicity in major depressive disorder: suggestive evidence for a bipolar contribution. J Affect Disord.

[63] Ripke S, Wray NR, Lewis CM (2013). A mega-analysis of genome-wide association studies for major depressive disorder. Mol Psychiatry.

[64] Wiste A, Robinson EB, Milaneschi Y (2014). Bipolar polygenic loading and bipolar spectrum features in major depressive disorder. Bipolar Disord.

[65] Musliner KL, Seifuddin F, Judy JA et al (2014) Polygenic risk, stressful life events and depressive symptoms in older adults: a polygenic score analysis. Psychol Med:1–12. 10.1017/S0033291714002839. 10.1017/S0033291714002839 PMC4412793 25488392

[66] Derks EM, Vorstman JAS, Ripke S (2012). Investigation of the genetic association between quantitative measures of psychosis and schizophrenia: a polygenic risk score analysis. PLoS One.

[67] Mullins N, Perroud N, Uher R (2014). Genetic relationships between suicide attempts, suicidal ideation and major psychiatric disorders: a genome-wide association and polygenic scoring study. Am J Med Genet B Neuropsychiatr Genet.

[68] Nievergelt CM, Maihofer AX, Mustapic M (2015). Genomic predictors of combat stress vulnerability and resilience in U.S. marines: a genome-wide association study across multiple ancestries implicates PRTFDC1 as a potential PTSD gene. Psychoneuroendocrinology.

[69] Middeldorp CM, de Moor MHM, McGrath LM (2011). The genetic association between personality and major depression or bipolar disorder. A polygenic score analysis using genome-wide association data. Transl. Psychiatry.

[70] Terwisscha van Scheltinga AF, Bakker SC, van Haren NEM (2013). Genetic schizophrenia risk variants jointly modulate total brain and white matter volume. Biol Psychiatry.

[71] Whalley HC, Sprooten E, Hackett S (2013). Polygenic risk and white matter integrity in individuals at high risk of mood disorder. Biol Psychiatry.

[72] Walton E, Geisler D, Lee PH (2014). Prefrontal inefficiency is associated with polygenic risk for schizophrenia. Schizophr Bull.

[73] Hall M-H, Chen C-Y, Cohen BM (2015). Genomewide association analyses of electrophysiological endophenotypes for schizophrenia and psychotic bipolar disorders: a preliminary report. Am J Med Genet B Neuropsychiatr Genet.

[74] Holmes AJ, Lee PH, Hollinshead MO (2012). Individual differences in amygdala-medial prefrontal anatomy link negative affect, impaired social functioning, and polygenic depression risk. J Neurosci.

